# Inhibition of ATM reverses EMT and decreases metastatic potential of cisplatin-resistant lung cancer cells through JAK/STAT3/PD-L1 pathway

**DOI:** 10.1186/s13046-019-1161-8

**Published:** 2019-04-08

**Authors:** Mingjing Shen, Zhonghua Xu, Weihua Xu, Kanqiu Jiang, Fuquan Zhang, Qifeng Ding, Zhonghen Xu, Yongbing Chen

**Affiliations:** Department of Cardiothoracic Surgery, The Second Affiliated Hospital of Soochow University, Suzhou, Jiangsu 215004 People’s Republic of China

**Keywords:** ATM, JAK_1,2_/STAT_3_, PD-L1, EMT, Cisplatin-resistant lung cancer

## Abstract

**Background:**

The cisplatin-resistance is still a main course for chemotherapy failure of lung cancer patients. Cisplatin-resistant cancer cells own higher malignance and exhibited increased metastatic ability, but the mechanism is not clear. In this study, we investigated the effects of Ataxia Telangiectasia Mutated (ATM) on lung cancer metastasis.

**Materials and methods:**

Cisplatin-resistant A549CisR and H157CisR cell line were generated by long-term treating parental A549 and H157 cells (A549P and H157P) with cisplatin. Cell growth, cell migration and cell invasion were determined. Gene expressions were determined by Western Blot and qPCR. Tumor metastasis was investigated using a xenograft mouse model.

**Results:**

The IC50 of the cisplatin-resistant cells (A549CisR and H157CisR cells) to cisplatin was 6–8 higher than parental cells. The A549CisR and H157CisR cells expressed lower level of E-cadherin and higher levels of N-cadherin, Vimentin and Snail compared to the parental A549P and H157P cells, and exhibited stronger capabilities of metastatic potential compared to the parental cells. The ATM expression was upregulated in A549CisR and H157CisR cells and cisplatin treatment also upregulated expression of ATM in parental cells, The inhibition of ATM by using specific ATM inhibitor CP466722 or knock-down ATM by siRNA suppressed Epithelial-to-Mesenchymal transition (EMT) and metastatic potential of A549CisR and H157CisR cells. These data suggest that ATM mediates the cisplatin-resistance in lung cancer cells. Expressions of JAK_1,2,_、 STAT_3_ 、PD-L1 and ATM were increased in A549CisR and H157CisR cells and could by induced by cisplatin in parental lung cancer cells. Interestedly, ATM upregulated PD-L1 expression via JAK_1,2_/STAT_3_ pathway and inhibition of ATM decreased JAK/STAT3 signaling and decreased PD-L1 expression. The treatment of PD-L1 neutralizing Ab reduced EMT and cell invasion. Inhibition of JAK_1,2_/STAT_3_ signaling by specific inhibitors suppressed ATM-induced PD-L1 expression, EMT and cell invasion. Importantly, inhibition of ATM suppressed EMT and tumor metastasis in cisplatin-resistant lung cancer cells in an orthotopic xenograft mouse model.

**Conclusions:**

Our results show that ATM regulates PD-L1 expression through activation of JAK/STAT3 signaling in cisplatin-resistant cells. Overexpression of ATM contributes to cisplatin-resistance in lung cancer cells. Inhibition of ATM reversed EMT and inhibited cell invasion and tumor metastasis. Thus, ATM may be a potential target for the treatment of cisplatin-resistant lung cancer.

**Electronic supplementary material:**

The online version of this article (10.1186/s13046-019-1161-8) contains supplementary material, which is available to authorized users.

## Mini abstract

Collectively, our findings established ATM as a potential indicator of outcome and drug responsiveness in lung cancer and inhibition of ATM may provide a novel choice in the overcome of tumor metastasis.

## Background

Lung carcinoma is the predominant cause of cancer death both in China and worldwide [1]. Lung cancer is mainly divided into non-small cell lung carcinoma (NSCLC) and small cell lung carcinoma (SCLC). NSCLC contributes to majority of 85% of lung carcinoma cases possessing its own biological characteristics and constitutes a heterogeneous population of adenocarcinoma, squamous and large cell carcinomas [2]. Platinum-based drugs, particularly cis-diammine-dichloroplatinum (II) (cisplatin, DDP), are widely used in clinics. Cisplatin has been demonstrated to be an effective drug for lung carcinoma treatment effect, but it will develop drug-resistance later on [3, 4].

We have previously found that ataxia telangiectasia mutated (ATM), a member of the phosphatidylinositol 3-kinase-related kinase family of Ser/Thr protein kinases, was induced by accumulated stimulation of cisplatin and was overexpressed in cisplatin-resistant NSCLC. Suppression of ATM expression could enhance the sensitivity of NSCLC to cisplatin treatment through activation of Erk, Akt, and MAPK pathways. Accumulated data also showed that chemo-resistance development is correlated with EMT process [5]. Cisplatin resistance in gastric cancer cells is associated with HER2 upregulation-induced epithelial-mesenchymal transition [6]. Furthermore, inhibition of EMT could overcome drug resistance in many types of cancers [7]. The results indicate that EMT is associated with development of drug-resistance.

Recent studies showed that DNA damage promotes chemo-resistance and drives EMT in colorectal carcinoma [8]. It is reported that Wip1 suppress ovarian cancer metastasis through inhibition of ATM/AKT/Snail pathway [9]. Singh [10] et al. found that ATM could mediate EMT in breast cancer. Several studies have shown that ATM expression is associated with EMT and metastatic potential of cancer cells. However, a report that loss of ATM accelerate EMT in pancreatic cancer [11]. Thus, the relationship between ATM and EMT and their roles in cisplatin-resistant NSCLC is still unclear. Based on the information above, we suppose the key molecule of DNA damage response (DDR), ATM not only contribute to cisplatin resistance but also play an important role in epithelial-mesenchymal transition (EMT) progress and tumor metastasis.

In this study, we investigated the roles of ATM and EMT in cisplatin-resistance and cancer metastasis in lung cancer cells. We found that overexpression of ATM in cisplatin resistant NSCLC is correlated with the process EMT and further elucidated a novel mechanism of ATM in mediating EMT and metastatic increase in cisplatin-resistant NSCLC preliminarily both in vitro and in vivo. This study will explain and mimic clinical significance since increased invasive features of cisplatin-resistant NSCLC have reported [12, 13].

## Materials and methods

### Cell culture

Lung cancer A549 and H157 cells were purchased from the American Type Culture Collection (ATCC, Manassas, VA) and cultured in RPMI 1640 containing 10% FBS. All cells were maintained in a humidified 5% CO2 environment at 37 °C. For inhibition studies, JAK inhibitor 1 (5 μM) (Calbiochem, CAS457081–03-7), Stattic (10 μM) (Calbiochem, CAS19983–44-9), and PD-L1 neutralizing Ab (10 μg/ml) (Invitrogen, 16–5982-82) that inhibits the ATM, JAK1,2, STAT3, and PD-L1 pathways, respectively, were added into the culture.

### Induction of cisplatin-resistant lung cancer cell lines

Parental A549 and H157 cells were continuously treated with gradually increased dose of cisplatin (0.5 mg/ml saline stock solution, Sigma) for 6 months according to method described by Barr et al. 4. Briefly, cells were treated with 1 μM cisplatin for 72 h and cells were allowed to recover for the following 72 h. After repeating one more cycle at 1 μM cisplatin concentration, the cells were then treated with 2 μM cisplatin in the following two cycles. This procedure was continued with increasing cisplatin concentration up to 30 μM. During the cisplatin-resistance induction procedure, the IC50 values of every 5 passage cells were accessed in comparison with those of the parental cells until the IC50 value remained constant. The cisplatin-resistant cell lines obtained by this method were maintained in growth media containing 10 μM cisplatin.

### Generation of ATM knocked-down and overexpression cell lines by lentiviral transduction

Lentivirus constructs carrying either ATMsiRNA or ATMRNA, or scramble sequence (pLenti-II vector, Addgene) were transfected into 293 T cells with a mixture of pLent-II-ATMsiRNA, psPAX2 (virus-packaging plasmid), and pMD2G (envelope plasmid) (4:3:2 ratio) using PolyFect Transfection reagent (Qiagen, Valencia, CA). These virus were harvested and storage for use. ATMsiRNA and ATMRNA, or sc virus infected After A549 and H157 cells overnight, the culture media containing the virus was replaced with normal culture media 48 h after infection, and maintained under normal cell culture conditions. After sub-culturing cells, the ATM knocked-down cells, overexpressing cells and sc cells were selected in the presence of puromycin (2 μg/ml) (Sigma) and then maintained in media containing 0.1 μg/ml puromycin. Stable cells were identified by determining the expression of ATM using Western blot.

### MTT assay

Cisplatin-cytotoxicity was analyzed by MTT (3-[4,5-dimethylthiazol-2-yl]-2,5-dipheny ltetrazolium bromide, 5 mg/ml, Sigma, USA) assay. Cells were seeded on 96-well plates (7 × 103 cells/well) and treated with various concentrations of cisplatin for 48 h. MTT test was then performed and absorbance at 490 nm was measured. Cell viability was calculated using the formula: OD sample/OD blank control × 100. Triplicate experiments were performed and average values with mean ± SEM were represented.

### Wound healing assay

Tumor cells (A549P/cisR, H157P/cisR cells and their control cells) were subjected to an in vitro wound assay with images captured at 12 h after using a microscope. Cells were seeded onto 6-well plates. When the cells reached 90–100% density, cells were scratched with 10□L sterile pipette tips. Cells were washed with PBS three times to remove detached cells and medium was replaced by fresh serum-free medium. The rate of migration was measured by quantifying the distance that cells moved from the edge of scratch toward the center of the scratch.

### Transwell assay

Tumor cells (A549P/cisR, H157P/cisR cells, either A549sc/siATM and H157sc/siATM cells, or inhibitor treated cells, 1 × 104, in serum-free media) were plated in upper chamber of transwell plates and 10% FBS-containing media (as chemotactic factor) was added in bottom wells. Before the assay, the membranes were pre-coated with 8% Matrigel. The Cells were cultured for 24 h. Invaded cells at the end of 24 h of incubation were visualized by staining with a crystal violet solution and counted under a microscope. Three independent experiments (with triplicates each experiment) were done and average numbers of positively stained cells in three randomly picked areas were presented in quantitation.

### In vivo mice studies

The luciferase tagged A549P, A549CisR cells and control cells (1 × 10^6^) that were obtained by transfection of luciferase reporter gene and selection procedure were orthotopically injected through pleural (1 × 10^6^ cells in media with Matrigel, 1:1 ratio in volume) into 8 weeks old female nude (National Cancer Institution, NCI). Tumor growth was monitored once a week by in vivo Image System (IVIS) with luciferin injection. When luminescence reached to 5 × 10^5^ to 1 × 10^6^ radiance(p/sec/cm2/sr), which corresponding to tumor size of 300-400 mm3, the A549cisR cells-inoculated mice were intraperitoneally (*i.p.*) injected with CP466722 (10 mg/kg) or vehicle (10%DMSO) every day. Tumor metastasis was monitored by IVIS once a week. After 8 weeks monitoring the metastasis, mice were sacrificed by euthanasia, the tumors were taken and fixed by formalin.

### RNA extraction and quantitative real-time PCR (qPCR) analysis

Total RNA (1 μg) was subjected to reverse transcription using Superscript III transcriptase (Invitrogen). qPCR was conducted using the appropriate primers and a Bio-Rad CFX96 system with SYBR green to determine the mRNA expression levels of genes of interest using the following protocol: 95 °C for 30 s, followed by 40 cycles of 95 °C for 5 s, 55 °C for 30 s, and 72 °C for 30 s. Each sample was detected in triplicate. Expression levels were normalized to (glyceraldehyde-3-phosphate dehydrogenase, GAPDH) level. The primer sequences for ATM, E-cad, N-cad, Snail, Vimentin, JAK1, JAK2, STAT3 and GAPDH were designed as follows: ATM sense primer 5′-CAGGGTAGTTTAGTTGAGGTTGACAG-3′,antisense primer 5′-CTATACTGGTGGTCAGTGCCAAAGT-3′.E-cad sense primer 5′-CAGAAAGTTTTCCACCAAAG-3′,antisense primer 5′-AAATGTGAGCAATTVTGCTT-3′.N-cad sense primer 5′-AGCCTGACACTGTGGAGCCT-3′,antisense primer 5′-TCAGCGTGGATGGGTCTTTC-3′. Snail sense primer 5′-GAGGCGGTGGCAGACTAGAGT-3′,antisense primer 5′-CGGGCCCCCAGAATAGTTC-3′.Vimentin sense primer 5′-GGCTCAGATTCAGGAACAGC-3′,antisense primer 5′-GCTTCAACGGCAAAGTTCTC-3′.JAK1 sense primer 5′-ACCGAGGACGGAGGAAAC-3′,antisense primer 5′-ACTGCCGAGAACCCAAAT-3′.JAK2 sense primer 5′-CAGCAGCTTGGCAAAGGTAACTTC-3′,antisense primer 5′-TCAGTGCTGTGCTGGAGTTTCTTC-3′.STAT3 sense primer 5′-CAGAAAGTGTCCTACAAGGGCG-3′,antisense primer 5′-CGTTGTTAGACTCCTCCATGTTC-3′.PD-L1 sense primer 5′-TATGGTGGTGCCGACTACAA-3′,antisense primer 5′-TGGCTCCCAGAATTACCAAG-3′.GAPDH sense primer 5′-CTCCTCCACATTTGACGCTG-3′,antisense primer 5′-TCCTCTTGTGCTCTTGCTGG-3′.A melting curve analysis was performed to monitor PCR product purity and the 2 − ΔΔCt method was used to quantify the expression of these indicated genes.

### Western blot analysis

Cells were lysed in RIPA buffer (50 mM Tris-Cl at pH 7.5, 150 mM NaCl, 1% NP-40, 0.5% sodium deoxycholate, 1 mM EDTA, 1 μg/mL leupeptin, 1 μg/mL aprotinin, 0.2 mM PMSF) and proteins (20–40 μg) were separated on 8–10% SDS/PAGE gel and then transferred onto PVDF membranes (Millipore, Billerica, MA). After blocking procedure, membranes were incubated with primary antibodies (1:1000), HRP-conjugated secondary antibodies (1:5000), and visualized in Imager (Bio-Rad) using ECL system (Thermo Fisher Scientific, Rochester, NY). Antibodies of ATM, p-ATM, JAK1, p-JAK1, JAK2, p-JAK2, STAT3, and p-STAT3, were from Gene Tex (Irvine, CA), Antibodies of E-cadherin, N-cadherin, Vimentin, Snail, Zeb1, Twist and VEGF were obtained from Abgent (San Diego, CA) and antibodies of PD-L1, GAPDH, were from Cell Signaling (Danvers, MA).

### Immunofluorescent analysis

Cells were cultured on coverslips in a 24-well plate, fixed with 4% paraformaldehyde for 15 min and permeabilized with 0.5% Triton X-100 solution for 5 min at room temperature. Then the coverslips were blocked with 5% bovine serum albumin in Tris buffered saline with Triton X-100 for 1 h and incubated with rabbit anti-E-cad, rabbit anti-N-cad (Cell Signaling Technology) and mouse anti-Snail (Abcam Technology) primary antibodies overnight at 4 °C. After being washed with phosphatebuffered saline three times, the coverslips were incubated with FITC conjugated anti-rabbit IgG and Cy3-conjugated anti-mouse IgG secondary antibodies (Biosharp, Shanghai, China) for 1 h at room temperature. Finally, cells were labeled with 4′-6-diamidino-2-phenylindole (Beyotime) and examined using a confocal laser scanning microscopy (LSM700, Zeiss, Oberkochen, Germany).

### Histology and immunohistochemistry

Tissues obtained were fixed in 10% (*v*/v) formaldehyde in PBS, embedded in paraffin, and cut into 5-μm sections. Tumor tissue sections were deparaffinized in xylene solution, rehydrated, and immunostaining was performed using the IHC kit (Santa Cruz, Santa Cruz). Antibodies of E-cadherin, N-cadherin, Vimentin, Snail (Cell Signaling, Danvers, MA), and ATM (Bethyl Laboratories, Montgomery, TX) (all antibodies at 1:250 dilution) were applied in staining. For ATM staining, the antigen retrieval process was performed in 10 mM Citric buffer, pH 6.0 for 20 min using a pressure cooker prior to staining. After staining, tissues were counterstained by Hematoxylin. Three microscopic visions were picked by random, and positively stained cells were determined.

### Statistics

The data values were presented as the mean ± SEM. Differences in mean values between two groups were analyzed by two-tailed Student’s t test. *P* ≤ 0.05 was considered statistically significant.

## Results

### EMT and metastatic potential is higher in cisplatin-resistant NSCLC cells than parental cells

**Fig. 1:**
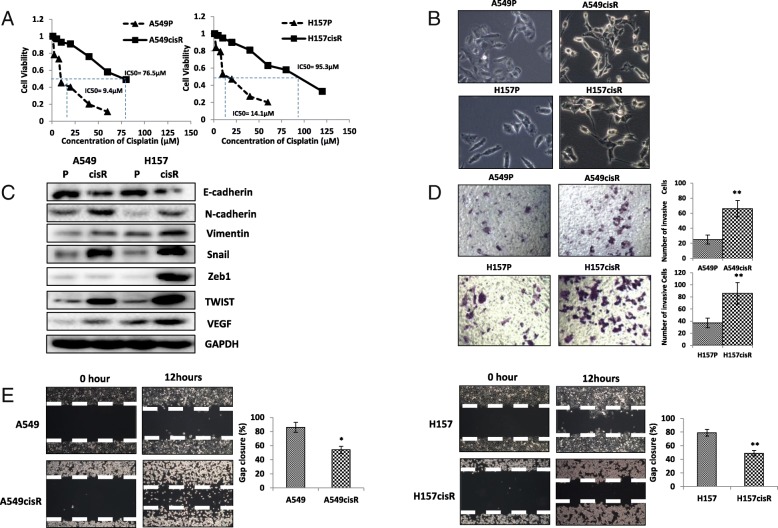
EMT and invasion potential are increased in cisplatin-resistant NSCLC cells. **a** Generation of cisplatin-resistant NSCLC cells. A549CisR and H157CisR cells were obtained by continuous treatment of A549P and H157 parental cells with increasing dose of cisplatin for 6 months. The IC50 of A549CisR and H157CisR and parental cells to cisplatin were analyzed in MTT assay. **b** Morphology alteration of cisplatin-resistant NSCLC cells. A549P/H157CisR H157P/H157CisR (1 × 10^3^) were cultured in normal media for 24 h and photos of cell morphology were obtained under microscope. **c** EMT alteration of cisplatin-resistant NSCLC cells. Total cell protein extracts were obtained from parental (A549P and H157P) and cisplatin-resistant cells (A549CisR and H157CisR) and Western blot analysis was performed using EMT-related antibodies indicated. **d** Increased Invasion potential in cisplatin-resistant NSCLC cells. Cells (A549P/A549CisR and H157P/H157CisR, 1 × 10^4^) were placed in upper chamber of transwell plates (8 μM pore) and invaded cells to lower chamber were counted under microscope 24 h after incubation. Photos of invade cells were taken (left panel) and Quantitation of invade cells was shown on right panel. **e** Increased Migration potential in cisplatin-resistant NSCLC cells. Cells indicated were seeded on 6-well plates and wound was made by scratch with 10uL tip. Closure distance was measured at 0 and 12 h after scratching. Quantitation analysis was shown on right panel. *p < 0.05, **p < 0.01, ***p < 0.001, compared to parental cells

We developed two cisplatin-resistant NSCLC cell lines, A549cisR and H157cisR, by treating A549P and H157P cells with a gradually increasing dose of cisplatin over 6 months [14]. These resistant cells showed almost 5 times higher IC_50_ values than parental cells (Fig. 1a).

We observed significant increased EMT and metastatic potential in A549cisR and H157cisR cells compared to their parental cells. Morphology studied showed that A549cisR and H157cisR cells exhibited mesenchymal phenotype [15] and showed the decreased ability of adherercance (Fig. 1b). Then, we found that expressions of EMT-related genes [15], including N-cadherin (N-cad), Vimentin, Snail, Zeb1, Twist, and vascular endothelial matrix growth factor (VEGF), was markedly increased in A549cisR and H157cisR cells compared to their parental cells. Meanwhile, expression of E-cadherin (E-cad) was decreased in A549cisR and H157cisR cells (Fig. 1c). Furthermore, invasion abilities were stronger in A549cisR and H157cisR cells than that in parental cells, which was consistent to EMT-related gene expressions (Fig. 1d). Accordingly, migration ability was significantly increased in A549cisR and H157cisR cells compared to the parental cells (Fig. 1e).

### Up-regulation of ATM in cisplatin-resistant NSCLC cells and short-time cisplatin treated parental cells

**Fig. 2:**
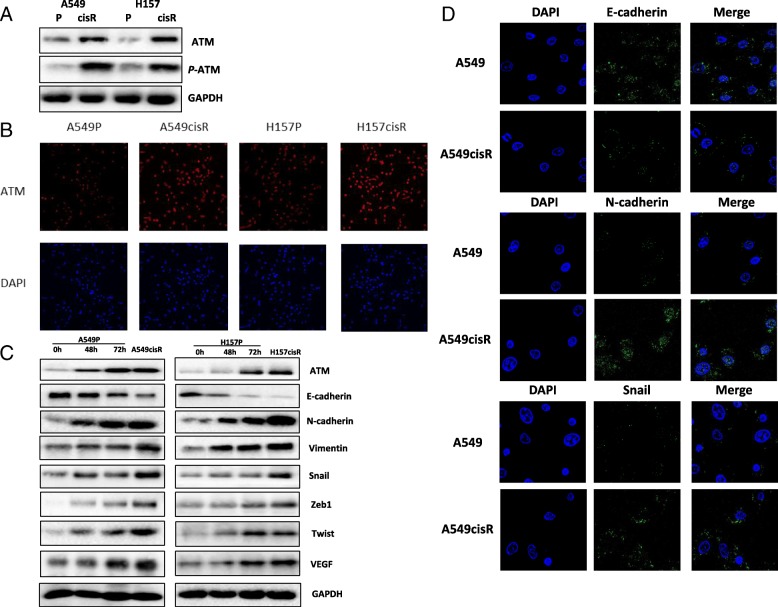
Increased ATM signaling in cisplatin-resistant NSCLC cells. **a** Increased ATM expression in cisplatin-resistant NSCLC cells. Total cell extracts were obtained from 549P/ A549CisR and H157P/H157CisR cells. Protein expressions were determined by Western blot analyses using antibodies indicated. **b** ATM localization in cisplatin-resistant NSCLC cells. Cells (A549P/A549CisR and H157P/ H157CisR) were stained with anti-ATM antibody and then stained with fluorescence- labelled second antibody. **c** Cisplatin treatment induces EMT in NSCLC cells. A549P and H157P cells were treated with 5 μM cisplatin for indicated time and cell extracts obtained. The expressions of EMT-related genes were determined by Western Blot using antibodies indicated. Represented images were shown. **d** Increased ATM expression is positively associated with EMT alteration of cisplatin-resistant NSCLC cells. Total cell protein extracts were obtained from parental (A549P and H157P) and cisplatin-resistant cells (A549CisR and H157CisR) and IFC was performed using EMT-related antibodies indicated

Cisplatin treatment can induce DNA lesions, leading to DNA repair, cell cycle arrest, senescence and apoptosis. These phenomena is called DNA damage repair (DDR) collectively. Our previous study [16] have already shown a series of molecular alterations associated with DDR pathway including ATM, DNA-PKcs, (PARP)-1, Ku70, BRCA1, bcl-2 which are highly expressed in cisplatin-resistance NSCLC. Among these molecules indicated, the level of ATM expression was dramatically higher in A549cisR and H157cisR cells than in their parental cells (Fig. 2a-b).

To investigate whether the increased expression of ATM in cisplatin-resistant cells is due to accumulated effect of cisplatin treatment, we treated A549 parental cells and H157 parental cells with short-term cisplatin treatment and found that the ATM levels were increased in A549 parental cells and H157 parental cells after treatment of cisplatin (5 μM) from 48 h to 72 h. Accordingly, EMT-related genes including E-cadherin, N-cadherin, Vimentin, Twist, Zeb1, VEGF and Snail were altered in those parental cells after short-term cisplatin treatment (Fig. 2c). These data demonstrate that cisplatin treatment can induce expression of ATM and EMT-related genes. To confirm the relationship between ATM and EMT-related genes expressions in parental and cisplatin-resistant cells, we picked up E-cad, N-cad, and Snail as representative gene scaled by IFC (Fig. 2d), which is consistent with the data in Fig. 1c.

### Inhibition of ATM expression results in significant reduction in EMT and metastatic potential in cisplatin-resistant NSCLC cells

**Fig. 3:**
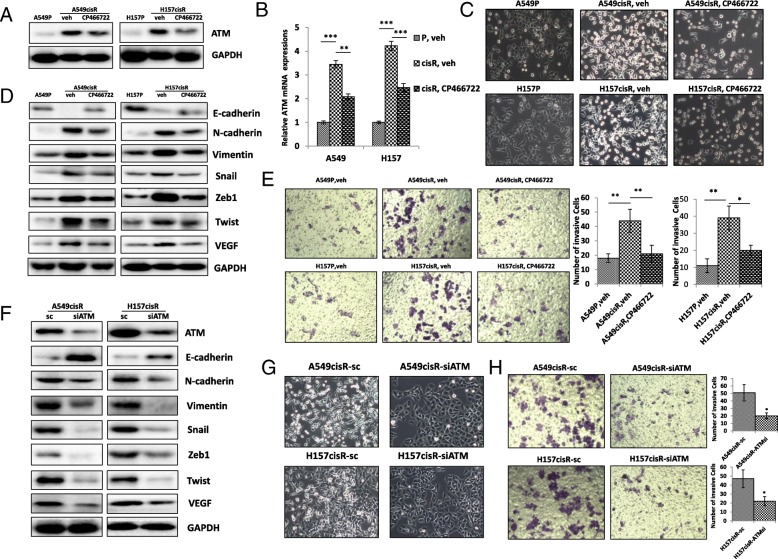
Inhibition of ATM reverses EMT and decreases invasion ability in cisplatin-resistant NSCLC cells. **a**-**b** ATM inhibitor (CP466722) decreased expression in cisplatin-resistant cells determined by Western blot analysis (**a**) and Real-time PCR analysis (**c**) Alteration in morphology in in cisplatin-resistant NSCLC cells. A549CisR and H157CisR cells were treated with ATM inhibitor for 24 h and cell morphology was taken under microscope. **d** The effects of ATM inhibitor on expressions of EMT-related genes in cisplatin-resistant NSCLC cells. A549CisR, H157CisR and parental cells were treated with ATM inhibitor for 24 h. Total cell extracts were obtained and protein expressions were determined by Western blot using antibodies indicated. Presented images were shown. **e** ATM inhibitor decreases cell invasion in cisplatin-resistant NSCLC cells. Cells indicated were incubated with ATM inhibitor in upper chamber of transwell plates (8 μm pore) and invasive cells to lower chamber were counted under microscope 24 h of incubation. Quantitation of invasion was shown on right panel. **f** Knockdown of ATM reverses the phenotype of EMT in in cisplatin-resistant NSCLC cells. Total cell extracts were obtained from A549CisR-sc/siATM, H157CisR-sc/siATM and control cells. Protein expressions were determined by Western blot analyses using antibodies indicated. **g** Alteration of cell morphology. A549CisR-sc/siATM and H157CisR-sc/siATM cells were cultured for 24 h and represented images of cell morphology were shown. **h** Knockdown of ATM reduced cell invasion. Cells indicated were seeded into upper chamber of transwell plates (8 μM pore) and invasive cells to lower chamber were counted under microscope 24 h after incubation. Quantitation analyses were shown on right Panel. *p < 0.05, **p < 0.01, ***p < 0.001, compared to control cells

To investigate whether ATM inhibition can affect the EMT and metastatic potential of cisplatin-resistant NSCLC cells, we treated A549cisR and H157cisR with an ATM inhibitor CP466722. As shown in Fig. 3a-b, ATM expression level was obviously decreased after CP466722 treatment, which was consistent with our previous results [16]. Interestingly, morphology change from mesenchymal-epithelial transition (MET) was also observed in A549cisR and H157cisR after CP466722 treatment (Fig. 3c). The expression of EMT-related genes were markedly decreased in A549cisR and H157cisR cells after CP466722 treatment (Fig. 3d). Consistently, invasion abilities in A549cisR and H157cisR cells were significant weak after CP466722 treatment (Fig. 3e). When we further decreased ATM expression in parental cells, no significant alternation of cell invasion and EMT were observed due to low expression of ATM in parental cells (Additional file 1: Figure S1A).

To further investigate the role of ATM in mediating cisplatin resistance, we generated the ATM knocked-down A549cisR and H157cisR cells (A549cisR-siATM and H157cisR-siATM) and control cells (Fig. 3f). The expressions EMT-related genes were significant decreased or increased in theA549cisR-siATM and H157cisR-siATM cells compared to control cells (Fig. 3f). These siATM cells showed morphology changes which were similar to the parental cells (Fig. 3g). Accordingly, the siATM cells exhibited the decreased of invasion ability than control cells (Fig. 3h). These results suggest that ATM plays a critical role in mediating EMT and metastasis in cisplatin-resistant NSCLC cells.

### ATM enhances EMT and metastatic potential via up-regulate PD-L1 in cisplatin-resistant NSCLC cells

**Fig. 4:**
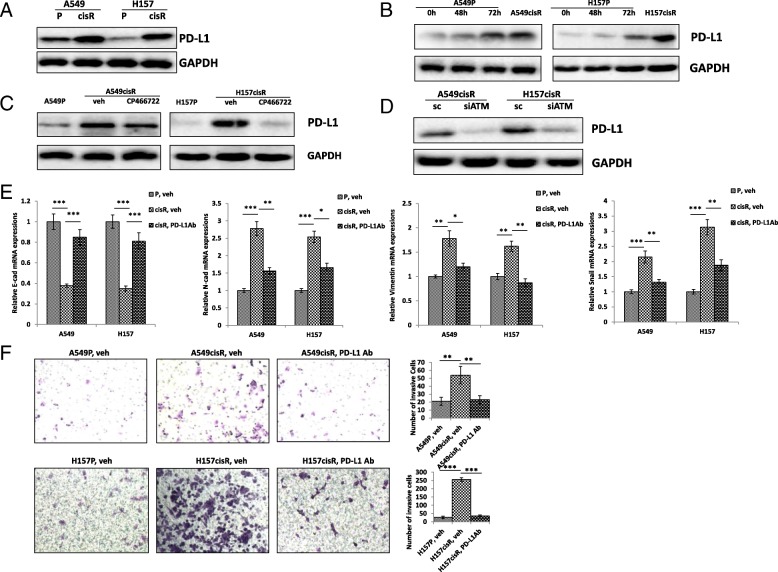
Upregulation of PD-L1 is related to EMT and invasion ability in cisplatin-resistant NSCLC. **a**. Higher expression of PD-L1 in cisplatin-resistant A549CisR and H157CisR cells determined by Western blot analysis. **b** Cisplatin treatment induces PD-L1 expression in NSCLC. A549P and H157P cells were treated with cisplatin (5 μM) for indicated time. Expression of PD-L1 was determined by Western blot. **c** ATM inhibitor (CP466722) downregulates PD-L1 expression. A549CisR and H157CisR cells were treated with inhibitor (CP466722) for 24 h. Expression of PD-L1 was determined by Western Blot. **d** ATM knockdown downregulates PD-L1 expression. A549CisR-siATM and H157CisR-siATM cells were generated Expression of PD-L1 was determined by Western Blot. **e** Anti-PD-L1 antibody inhibits phenotype of EMT in cisplatin-resistant cells. A549CisR and H157CisR cells were treated with PD-L1 neutralizing antibody for 24 h. Expressions of mRNA of EMT-related genes were determined by qPCR analysis. **f** Anti-PD-L1 antibody reduced cells invasion. A549P/A549CisR and H157P/H157CisR cells were placed in upper chamber of transwell plates (8 μM pore) and incubated with PD-L1 neutralizing antibody for 24 h. Invasive cells to lower chamber were counted under microscope 24 h after incubation. Quantitation analyses were shown on right panel. *p < 0.05, **p < 0.01, ***p < 0.001, compared with control treatment

To investigate the molecular mechanism by which ATM mediates cisplatin resistance, we investigated which is the downstream molecule of ATM that can mediate EMT and metastatic potential in cisplatin-resistant NSCLC cells. It has been reported that that PD-L1 is associated with EMT transition in adenocarcinoma of lung and in head and neck squamous cell carcinoma respectively [17, 18], we hypothesis that PD-L1 may play a role in mediated ATM/EMT pathway in cisplatin-resistant NSCLC cells. First, we investigated the PD-L1 expression in A549cisR and H157cisR cells and found that expression of PD-L1 levels was higher in A549cisR and H157cisR cells than in control parental cells (Fig. 4a). Accordingly, up-regulation of PD-L1 expression was detected in A549P and H157P after cisplatin treatment (Fig. 4b). Interestingly, ATM inhibitor (CP466722) reduced PD-L1 expression in A549cisR and H157cisR cells (Fig. 4c). Knockdown of ATM decreased expression of PD-L1 in A549cisR-siATM and H157cisR-siATM cells compare to sc cells (Fig. 4d), suggesting that ATM mediates PD-L1 expression in cisplatin-resistant lung cancer cells.

We then treated A549cisR and H157cisR cells with PD-L1 neutralizing Ab to determine whether PD-L1 signaling can inhibit EMT and metastatic potential. The results showed that the expression of EMT marker (E-cad) was increased and the expressions of N-cad, Vimentin and Snail were decreased after the PD-L1 neutralizing Ab treatment in A549cisR and H157cisR cells (Fig. 4e). Furthermore, the treatment of PD-L1 neutralizing Ab reduced the abilities of invasion in A549cisR and H157cisR cells (Fig. 4f). These results suggest that PD-L1 is the ATM downstream molecule that mediates EMT and metastasis of A549cisR and H157cisR cells.

### ATM up-regulate PD-L1 through JAK_1,2_/STAT_3_ pathway in cisplatin-resistant NSCLC cells

**Fig. 5:**
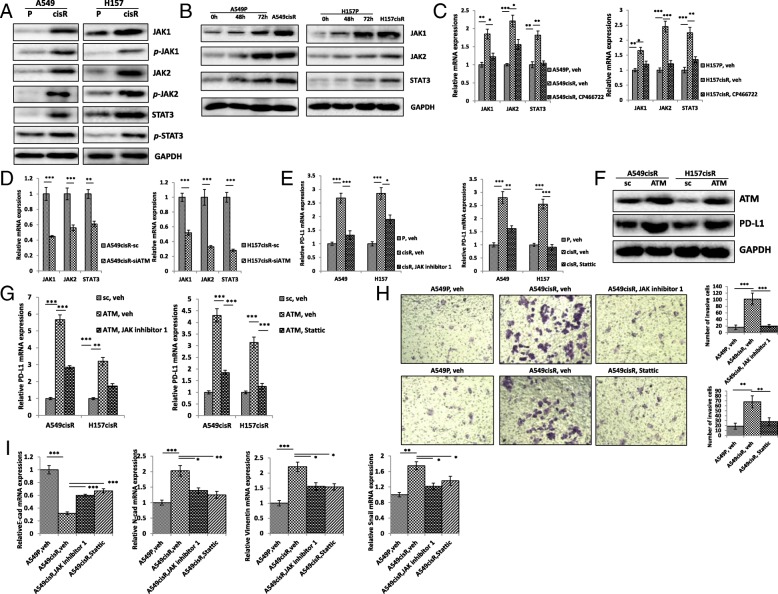
ATM up-regulate PD-L1 through JAK_1,2_/STAT_3_ pathway in cisplatin-resistant NSCLC cells. **a** Activation of JAK/STAT_3_ signaling in cisplatin-resistant NSCLC. Protein expressions of JAK_1,2_/STAT_3_ signaling molecules were determined by Western blot. **b** Cisplatin treatment induces JAK/STAT pathway in NSCLC cells. A549P and H157P cells were treated with 5 μM cisplatin for indicated time and cell extracts obtained. The expressions of JAK_1_, JAK_2_, and STAT_3_ were determined by Western Blot using antibodies indicated. Represented images were shown. **c** ATM inhibitor decreases mRNA expressions of JAK_1_, JAK_2_ and STAT_3_ determined by qPCR analysis in A549CisR and H157CisR cells. **d** ATM Knockdown decreases mRNA expression of JAK_1,2_/STAT_3_ determined by qPCR in A549CisR-siATM and H157CisR-siATM cells A549CisR. **e** JAK_1,2_ inhibitors JAK inhibitor 1 and STAT_3_ inhibitor stattic downregulates mRNA expressions of PD-L1 determined by qPCR analysis in A549CisR and H157CisR cells (**f**) ATM overexpression upregulates PD-L1 expression in A549CisR-ATM and H157CisR-ATM cells determined by Western blot. **g** JAK_1,2_ inhibitor JAK inhibitor 1 and STAT_3_ inhibitor stattic downregulates mRNA expressions of PD-L1 determined by qPCR analysis in in A549CisR-ATM and H157CisR-ATM cells. **h** JAK_1,2_ inhibitors JAK inhibitor 1 and STAT_3_ inhibitor stattic reduces cell invasion in cisplatin-resistant cells. A549CisR and H157P/H157CisR cells were seeded in upper chamber of transwell plates (8 μM pore) in the presentation of JAK inhibitor 1 and stattic, 1 × 10^4^) and invasive cells to lower chamber were counted 24 h after the treatment. Quantitation analysis was shown on right panel. **i** JAK inhibitor 1 and stattic affects mRNA expression of EMT-related genes determined by qPCR in A549CisR and H157CisR cells. *p < 0.05, **p < 0.01, ***p < 0.001, compared to sc cells

We observed that expressions of JAK_1_, *p-*JAK_1,_ JAK_2_, *p-*JAK_2_, STAT_3_, *p-*STAT_3_ were higher in A549cisR and H157cisR cells than in A549P and H157P cells, consistent with our previous study [16] (Fig. 5a). Accordingly, up-regulation of JAK_1_, JAK_2_ and STAT_3_ expression was detected in A549P and H157P after cisplatin treatment (Fig. 5b). In our another report [19], we observed inhibition of JAK_1,2_/STAT_3_ pathway obviously decreased PD-L1 expression in radiation resistant NSCLC along with ERK inhibitor, consistent with previous studies [20, 21] that upstream of JAK_1,2_/STAT_3_ pathway regulates. Furthermore, we found that treatment with ATM inhibitor CP466722 down-regulated expressions of JAK_1_, JAK_2_ and STAT_3_ in A549cisR and H157cisR cells (Fig. 5c). Similarly, the expression levels of JAK_1_, JAK_2_ and STAT_3_ was lower in A549cisR-siATM and H157cisR-siATM cells than in control parental cells (Fig. 5d).

Then, we treated A549cisR and H157cisR cells with JAK_1,2_ inhibitor (JAK inhibitor1), or STAT_3_ inhibitor (stattic), to determine the effects of JAK/STAT signaling on PD-L1 expression. The results showed that the treatment of JAK inhibitor1 and stattic decreased PD-L1 expression in the A549cisR and H157cisR cells (Fig. 5e). These data suggest that JAK_1,2_/STAT_3_ pathway is involved in ATM-regulating PD-L1 expression. To further elucidate whether ATM downregulates PD-L1 via JAK_1,2_/STAT_3_ pathway, we generated ATM-overexpression A549CisR and H157CisR cells (A549CisR-ATM and H157CisR-ATM) and confirmed that ATM-overexpression increased the expression of PD-L1 expression in lung cancer cells (Fig. 5f). We found that JAK inhibitor1 and stattic treatment recovered PD-L1 expression in A549CisR-ATM and H157CisR-ATM cells compared to the sc cells (Fig. 5g). The results suggest that ATM up-regulate PD-L1 through JAK_1,2_/STAT_3_ pathway in cisplatin-resistant NSCLC cells.

To determine whether JAK_1,2_/STAT_3_ pathway is associated with EMT and metastatic potential of lung cancer cells, we treat A549cisR and H157cisR cells with JAK inhibitor1 and stattic, respectively, and found that abilities of invasion were reduced (Fig. 5h). Consistent with invasion ability, expression of E-cad, was increased, and the expressions of N-cad, Vimentin and Snail were decreased in A549cisR and H157cisR cells after JAK inhibitor 1 and stattic treatment (Fig. 5i). These results suggest that ATM down-regulate PD-L1 through JAK_1,2_/STAT_3_ pathway that mediates EMT and metastasis in cisplatin-resistant A549cisR and H157cisR cells.

### Inhibition of ATM suppresses tumor metastasis in orthotopic xenograft mouse models

**Fig. 6:**
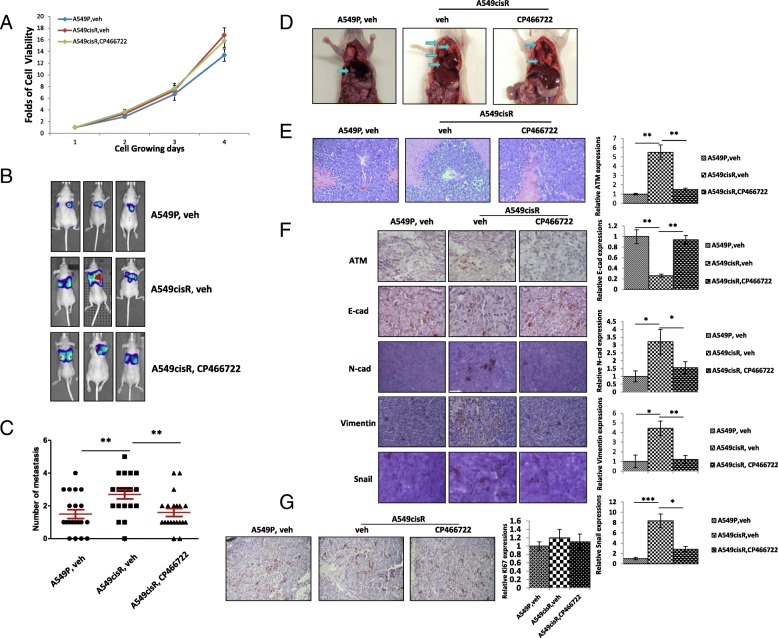
ATM inhibitor suppresses tumor metastasis in cisplatin-resistant NSCLC cells. **a** Cell death effect of CP compound in vitro. To show the effect of CP compound on animal model is not caused by cell death in vivo, we treated cells in vitro the CP466722 for long term treatment more than 72 h by MTT assay. **b**-**d** ATM inhibitor suppresses lung metastasis in cisplatin-resistant NSCLC cells. A549P and A549CisR cells were injected into lung through pleural. Tumor growth and metastasis were weekly monitored for 8 weeks by IVIS Images machine. Representative mice were taken 8 weeks after the injections and shown in (**b**). Numbers of tumor metastasis in each group was shown in (**c**). Representative imaging of tumor metastasis were presented in (**d**). **e** HE staining of metastatic tumors. A549P and A549CisR xenografts tumor tissues obtained from CP466722-treated mice or control mice were stained by HE, confirming the harvested the metastatic tumors. **f** ATM inhibitor (CP466722) suppresses EMT in vivo. A549P and A549CisR xenografts tumor tissues obtained from CP466722-treated mice or control mice were subjected to IHC staining using the antibodies anti-EMT-related genes. Represented images were shown in left panel. Quantitation of positively stained cells was shown on right panel. **g** Ki67 expression in vivo. A549P and A549CisR xenografts tumor tissues obtained from CP466722-treated mice or control mice were subjected to IHC staining using Ki67. *p < 0.05, **p < 0.01, ***p < 0.001, compared to the control treatment

To investigate whether ATM contributes to metastasis potential in cisplatin-resistant NSCLC cells, we determine the effects of ATM inhibitor on metastasis of lung cancer cells using a xenograft mouse model. Orthotopic xenograft mouse models were generated through pleural injecting luciferase-tagged A549P and A549cisR cells. Both in in vitro and in vivo experiments, luciferase-tagged A549P and A549cisR cells were either cultured with vehicle or CP466722 confirming CP466722 doesn’t influent tumor metastasis by inhibiting cell growths (Fig. 6a&g**)**. Tumor growth was monitored once a week by in vivo Image System (IVIS) with luciferin injection. When luminescence reached to 5 × 10^5^ to 1 × 10^6^ radiance(p/sec/cm^2^/sr), which corresponding to tumor size of 300-400 mm^3^, the A549cisR cells-inoculated mice were intraperitoneally *(i.p.*) injected with CP466722 (10 mg/kg). The control group mice (Either A549P cells or A549cisR cells) were injected with vehicle (10%DMSO) every day. Tumor metastasis was monitored by IVIS once a week. We found that mice injected with A549cisR cells exhibited stronger imaging in the lung than A549P cells (Fig. 6b). Quantitation analysis showed that the number of metastatic lung tumor was more in the A549cisR-injected mice than A549P-injected mice (Fig. 6c). We observed metastatic tumor in lung, mediastinal lymph node, abdominal cavity, bone and brain in the A549cisR-injected mice. The results indicate that A549cisR cells had stronger capacities of metastasis than A549P cells. Importantly, CP466722 treatment significantly reduced the number of metastatic tumor in the A549cisR-injected mice (Fig. B-D), suggesting that ATM play an important role in mediating tumor metastasis in cisplatin-resistant lung cancer cells. HE staining of tissue from each groups showed in Fig. E confirming they were metastatic tumors.

To determine the mechanisms by which ATM promotes tumor metastasis, we performed the IHC staining using xenografts tumor tissues obtained from A549P cell –injected and A549cisR cell-injected mice treated with CP466722 or vehicle. The results showed that expressions of ATM were increased in tumor issues of A549cisR cell-derived xenografts compared to tissues of A549P cell-derived xenografts (Fig. 6f), consisting with the in vitro result showing increased ATM expression level in cisplatin-resistant cells compared to the parental cells. In addition, we observed reduced ATM staining level in tumor tissues obtained from CP466722-treated A549cisR-incoulated mice compared to the vehicle-injected A549cisR-incoulated mice. Accordingly, E-cad expression were decreased in tumor issues of A549cisR cell-derived xenografts compared to tissues of A549P cell-derived xenografts (Fig. 6f), while the expressions of N-cad, Vimentin and Snail were higher in tissues of A549cisR cell-derived xenograft tumor than in A549P cell-derived xenografts tumor (Fig. 6f). Furthermore, the CP466722 treatment significantly increased expression of E-cad and decreased the expression of N-cad, Vimentin and Snail in the A549cisR cell-derived xenograft tumor (Fig. 6f). The results suggest that targeting inhibition of ATM can reverse EMT and metastasis potential in vivo in cisplatin-resistant NSCLC cells.

## Discussion

In our previous study, we found that increased ATM expression is related to cisplatin resistance formation, and knockdown ATM can enhance the sensitivity of cisplatin treatment in lung cancer cells. In this study, we further investigated the role of ATM in EMT and metastasis of lung cancer cells. Some studies have indicated that ATM expression is related to EMT and tumor metastasis in other tumor cells. Chen S [22] et al. reported that ATM was involved in EMT in pancreatic cancer by regulation of long non-coding RNA ANRIL. Liu R [23] et al. reported that depletion of ATM inhibited colon cancer proliferation and migration via Chk1/P53/CD44 cascades. In this study, we for the first time demonstrated that increased ATM expression contribute to increased EMT and metastatic potential in cisplatin-resistant NSCLC cells.

Studies have shown that ATM mainly functions as a central regulator of DNA damage response (DDR) [24], and help responding to heat stress, hypoxia, peroxide, inflammation and radiation [25]. So far, the mechanism by which DDR participates in drug resistance (such as cisplatin resistance) is not completely understood. While it is well known that cisplatin may inhibit DNA duplication and cause double strands breaking and crosslinking of DNA, resulting in impeding cell proliferation and inhibiting tumor growing. We further wonder whether there is any relationship between drug resistance and EMT as well as tumor metastasis. It has reported that a number of signaling pathways is involved both in chemo-resistance and EMT, including HER2-snail axis, MEK/ERK, Stat3 and AKT [6, 7, 9, 26] In addition, Kim HP [26] et al. showed that EMT signaling confers to acquired resistance to gastric cancer. Oliveras-Ferraros C [27] et al. reported that EMT confers to primary resistance to trastuzumab (Herceptin). EMT process functions as a driving factor contributing to drug resistance. It could be easy to understand that cisplatin-resistant NSCLC cells had more malignant phonotype not only showing drug resistance but also exhibiting potential metastatic ability. The clinic studies have shown that the patients with cisplatin-resistance had a poor curative effect and poor prognosis. In this study, our results showed that cisplatin-resistant lung cancer cells expressed high level ATM, and over-expressing lung cancer cells exhibited cisplatin-resistance and EMT. Thus, ATM not only contributes to drug resistance, but also is an important regulator of EMT and metastasis. But the molecular mechanism by which ATM mediates chemo-resistance, EMT and tumor metastasis is not fully understood.

Metastasis is the primary cause of death in cancers, especially in NSCLC and breast cancers [28]. Tumor metastasis is a compatible process starting with primary tumor cell invasion in which EMT plays a critical role. EMT is characterized by endothelial cell exhibiting mesenchymal phonotype by loss of cell-to-cell adhesion and increase of cell motility [28, 29]. In this study, first, we observed that cisplatin-resistant A549cisR and H157cisR cells showed a reduced cell-to-cell contact and decreased number of cell colony compared to parental A549P and H157P cells. We observed one interesting phenomena in which A549cisR and H157cisR cells spread more directions compared to the parental cells when these cells were cultured at low concentration of serum medium during the cell migration. This phenomenon indicates that cisplatin-resistant cell subclone own more migration character, especially in environment of infertile nutrition. This special phenotype is depending on the conditioned medium containing 10 mM cisplatin. The cell phenotype and EMT ability could be reversed if cisplatin-resistant cells have been cultured in cisplatin free medium for more than 4–6 weeks (Additional file 2: Figure S2A&B). Secondly, we found A549cisR and H157cisR cells possessed an increased ability of EMT accompanied by decreasing expression of E-cadherin and increasing expressions of N-cadherin, Vimentin, Twist and Snail. Previous studies showed activated Snail can bind to the E-boxes of E-cadherin inhibiting its expression at plasma membrane and ATM can stabilize Snail via phosphorylation at serine 100 [30, 31]. Our results showed that ATM inhibitor treatment in A549cisR and H157cisR cells or siRNA knock-down of ATM in A549cisR and H157cisR cells resumed epithelial cell phonotype, showing an increased cell-to-cell contact, round shape, and colony formation. In addition, increased E-cadherin and decreased N-cadherin, Vimentin, Twist, snail, Zeb were also observed in ATM inhibitor treated cells or A549cisR-siATM and H157cisR-siATM cells, suggesting that ATM inhibition can reverse EMT and metastasis. In addition, ATM inhibitor-treated A549cisR and H157cisR cells or A549cisR-siATM and H157cisR-siATM cells abrogated the increased ability of cell invasion. In summary, all above findings showed an essential role of ATM in EMT and metastasis in cisplatin-resistant cells.

It has been reported that ATM effects on EMT and metastatic potential through activation of JAK/STAT3 pathway [32]. Our previous results showed that continuous cisplatin treatment induce ATM expression and EMT [33]. Recent studies have shown that PD-L1 its function on promoting cell migration and invasion are gradually emphasized. Kim [17] et al. proved PD-L1 expression is associated with EMT in adenocarcinoma of lung. Wang Y [34] et al. reported PD-L1 induced EMT via activating SREBP-1c in renal cell carcinoma. PD-L1 (CD274) is regarded as an important mediator of T cell proliferation and PD-L1/PD1 signaling inhibits immune response and results in immune escape of tumor cell [35–37]. Studies have shown that PD-L1 expression can be induced by extrinsic stimulation such as interferon-gamma produced by surrounding tumor cells [38], and by the activation of intrinsic oncogenic pathways, such as STAT_3_, an activating EGFR mutation or ALK translocation [39, 40]. In this study, we found that ATM regulated PD-L1 expression via activation of JAK_1,2_/STAT_3_ pathway, consisting with previous reports [41]. JAK/STAT signaling activation, which is required for diverse process during embryogenesis and now, is thought to be associated with cancers. JAK(_S_) could be activated after an extracellular ligand binds to its receptor. The phosphorylated JAK/STAT proteins then dimerize and shuttle into the nucleus where they function as a transcription factor. STAT_3_ is regarded as one of the master regulators of EMT programs including twist, snail, slug, foxc2, zeb1 and zeb2. In this study we found that STAT_3_ signaling activation regulated PD-L1 expression in cisplatin-resistant lung cancer cells.

So far, no studies have revealed the detail mechanisms how ATM regulates PD-L1 expression through JAK_1,2_/STAT_3_ pathway. Zhang [42] et al. found that ATM was involved in phosphorylation of STAT3. Similarly, SUN [31] et al. reported that ATM stabilized Snail via phosphorylation at serine 100, and allowed Snail to down-regulate E-cadherin expression resulting in EMT finally. Since ATM protein is not a transcription factor, we wonder whether it may regulate EMT through downstream signaling molecules via mythelation, phosphorylation or ubiquitination. We found that ATM doesn’t regulate PD-L1 directly, but activate JAK/STAT3 signaling.

## Conclusions

In summary, ATM is highly expressed in cisplatin-resistant NSCLC and over-expression of ATM contributes to EMT via JAK_1,2_/STAT_3_-PD-L1 pathway. ATM exhibits multiple functions not only in mediating EMT, but also correlating with cisplatin-resistance. ATM may be a promising target for NSCLC treatment.

## Additional files

Additional file 1: **Figure S1.** (PDF 347 kb)

Additional file 2: **Figure S2.** (PDF 297 kb)

## Abbreviations

ATM: Ataxia Telangiectasia Mutated
DDR: DNA damage repair
EMT: epithelial-mesenchymal transition
HE: Hematoxylin-eosin
IFC: Immunofluorescence confocal
IHC: Immunohistochemistry
IVIS: In vivo Image System
JAK: Janus kinase
NSCLC: Non-small cell lung carcinoma
PD-L1: Programmed cell death 1 ligand-1.
STAT: Signal transducer and activator of transcription
VEGF: Vascular endothelial matrix growth factor
