# Supplementary figures and images for: Inhibition of ATM reverses EMT and decreases metastatic potential of cisplatin-resistant lung cancer cells through JAK/STAT3/PD-L1 pathway
Source: J Exp Clin Cancer Res. 2019 Apr 8;38:149. doi: 10.1186/s13046-019-1161-8 (PMC6454747; doi:10.1186/s13046-019-1161-8)

# Supplementary Figure 1

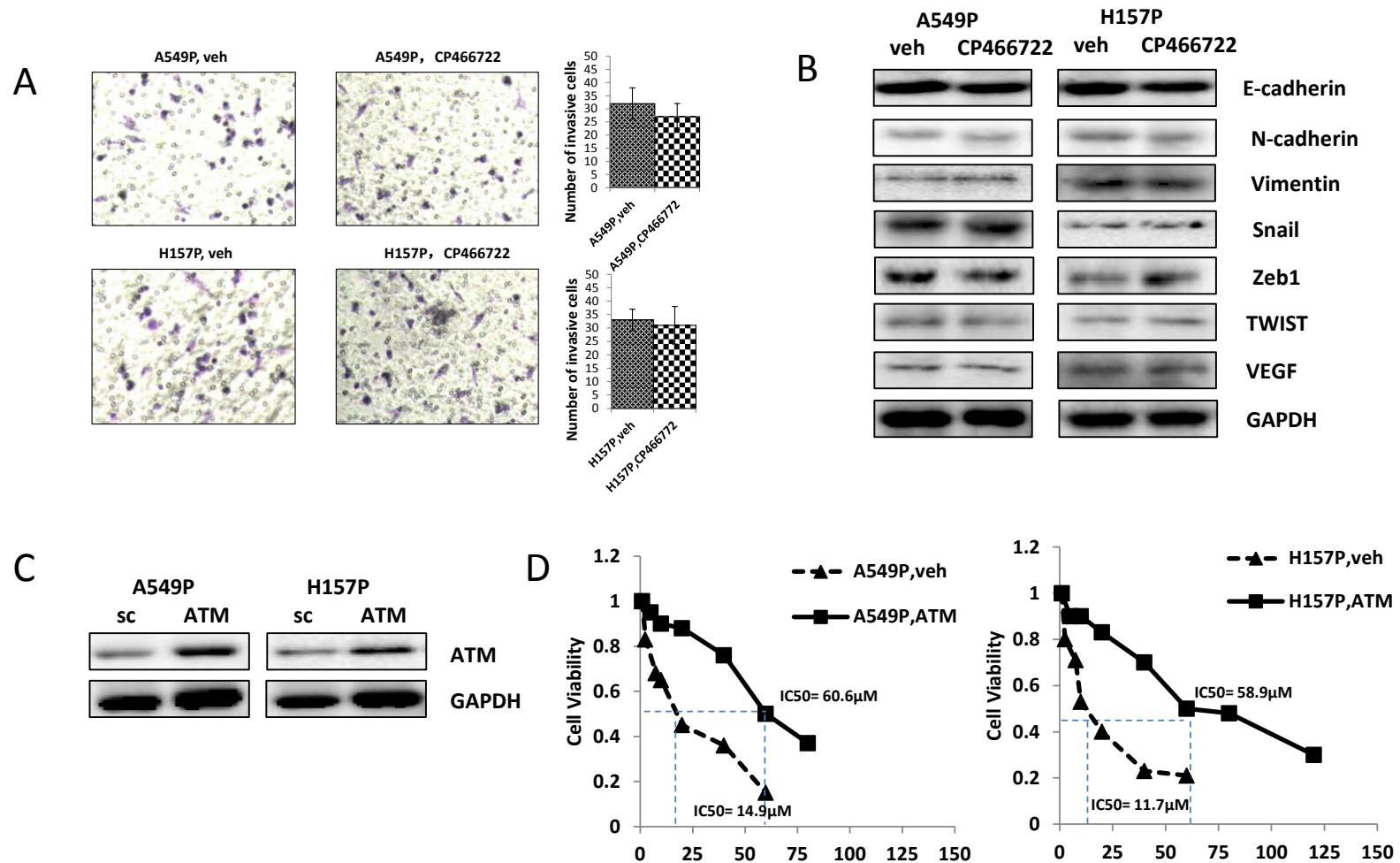

Supplement: Supplementary file 1 — Figure S1. (PDF 347 kb) [file 13046_2019_1161_MOESM1_ESM.pdf]

# Supplementary Figure 2

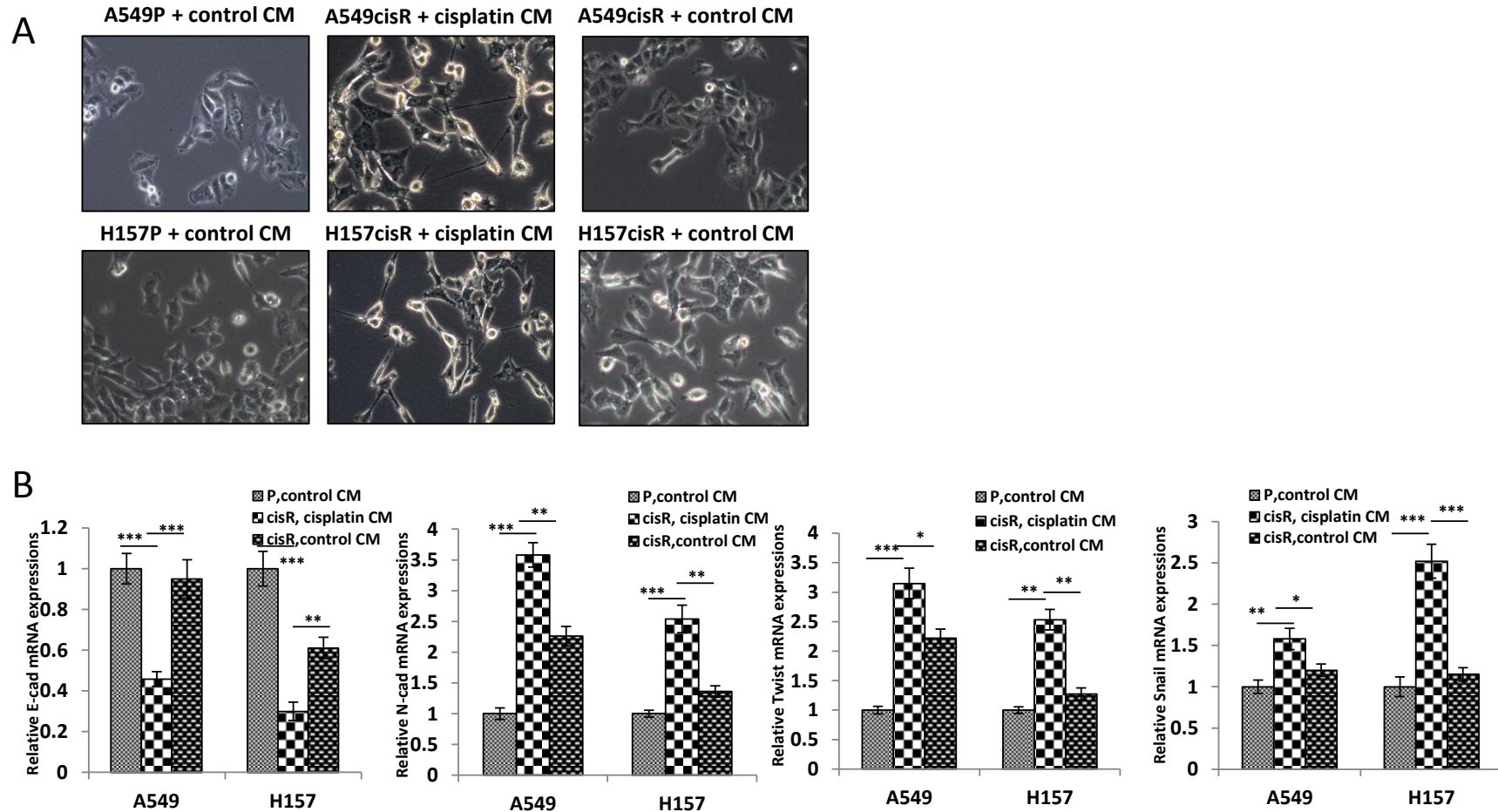

Supplement: Supplementary file 2 — Figure S2. (PDF 297 kb) [file 13046_2019_1161_MOESM2_ESM.pdf]
